# Liver-specific overexpression of lipoprotein lipase improves glucose metabolism in high-fat diet-fed mice

**DOI:** 10.1371/journal.pone.0274297

**Published:** 2022-09-13

**Authors:** Kahori Shimizu, Syogo Nishimuta, Yuri Fukumura, Shotaro Michinaga, Yuka Egusa, Tomomi Hase, Tomoyuki Terada, Fuminori Sakurai, Hiroyuki Mizuguchi, Koji Tomita, Toru Nishinaka

**Affiliations:** 1 Laboratory of Biochemistry, Faculty of Pharmacy, Osaka Ohtani University, Osaka, Japan; 2 Department of Pharmacodynamics, Meiji Pharmaceutical University, Tokyo, Japan; 3 Laboratory of Biochemistry and Molecular Biology, Graduate School of Pharmaceutical Sciences, Osaka University, Osaka, Japan; 4 Global Center for Medical Engineering and Informatics, Osaka University, Osaka, Japan; 5 Laboratory of Hepatocyte Differentiation, National Institutes of Biomedical Innovation, Health and Nutrition, Osaka, Japan; 6 Integrated Frontier Research for Medical Science Division, Institute for Open and Transdisciplinary Research Initiatives (OTRI), Osaka University, Osaka, Japan; 7 Laboratory of Molecular Biology, Faculty of Pharmacy, Osaka Ohtani University, Osaka, Japan; Max Delbruck Centrum fur Molekulare Medizin Berlin Buch, GERMANY

## Abstract

The liver is the main organ that regulates lipid and glucose metabolism. Ectopic lipid accumulation in the liver impairs insulin sensitivity and glucose metabolism. Lipoprotein lipase (LPL), mainly expressed in the adipose tissue and muscle, is a key enzyme that regulates lipid metabolism via the hydrolysis of triglyceride in chylomicrons and very-low-density lipoproteins. Here, we aimed to investigate whether the suppression level of hepatic lipid accumulation via overexpression of LPL in mouse liver leads to improved metabolism. To overexpress LPL in the liver, we generated an LPL-expressing adenovirus (Ad) vector using an improved Ad vector that exhibited considerably lower hepatotoxicity (Ad-LPL). C57BL/6 mice were treated with Ad vectors and simultaneously fed a high-fat diet (HFD). Lipid droplet formation in the liver decreased in Ad-LPL-treated mice relative to that in control Ad vector-treated mice. Glucose tolerance and insulin resistance were remarkably improved in Ad-LPL-treated mice compared to those in control Ad vector-treated mice. The expression levels of fatty acid oxidation-related genes, such as peroxisome proliferator-activated receptor α, carnitine palmitoyltransferase 1, and acyl-CoA oxidase 1, were 1.7–2.0-fold higher in Ad-LPL-treated mouse livers than that in control Ad-vector-treated mouse livers. Furthermore, hepatic LPL overexpression partly maintained mitochondrial content in HFD-fed mice. These results indicate that LPL overexpression in the livers of HFD-fed mice attenuates the accumulation of lipid droplets in the liver and improves glucose metabolism. These findings may enable the development of new drugs to treat metabolic syndromes such as type 2 diabetes mellitus and non-alcoholic fatty liver disease.

## Introduction

Body fat mass is determined by the balance between the storage and removal of adipose cell triglycerides (TGs) [1]. Adipose tissues store excess energy in the form of TGs and play an important role in energy homeostasis [2]. However, adipose tissue dysfunction leads to the accumulation of TGs in non-adipose tissues as ectopic fat [3–5]. Ectopic fat deposition interferes with cellular or organ functions and can occur in several organs and tissues such as muscles, the liver, and the pancreas. The liver is a major metabolic organ that plays an essential role in regulating lipid and glucose homeostasis. Lipid accumulation in the liver is strongly associated with the development of hepatic insulin resistance, which leads to abnormal glucose and lipid metabolism [6, 7]. Insulin resistance is typically defined as decreased sensitivity to the metabolic activity of insulin and is observed in patients with type 2 diabetes mellitus and obesity.

Excessive lipid accumulation in the liver represents a complex interaction among hepatic fatty acid uptake, *de novo* lipogenesis, fatty acid oxidation in mitochondria and peroxisomes, and export as TG within very low-density lipoprotein [8]. During hepatic fatty acid catabolism, free fatty acids are activated into their fatty acyl-CoA derivatives. The activated fatty acyl-CoAs are taken up into the mitochondria or peroxisome for degradation to acetyl-CoA via fatty acid oxidation [9]. Fatty acid activates the peroxisome proliferator-activated receptor α (PPARα), the main regulator of fatty acid oxidation [10], leading to enhanced fatty acid oxidation and adenosine triphosphate production.

The oxidation of hepatic fatty acid occurs primarily within mitochondria. Transport of free fatty acid inside the mitochondria matrix is controlled by a carnitine shuttle, composed of carnitine palmitoyltransferases 1 and 2 (CPT1 and CPT2) and carnitine acyltranslocase [8]. Previous studies reported an association between decreased mitochondrial function and insulin resistance. The activity of CPT1, a rate-limiting enzyme for controlling entry and oxidation of fatty acids [11], was reduced in the skeletal muscle, leading to decreased fatty acid oxidation in patients with obesity compared to those in lean control subjects [12]. Mitochondrial activity and size are decreased in the skeletal muscle of patients with type 2 diabetes mellitus and obesity [13]. Citrate synthase, the first enzyme of the citric acid cycle involved in the irreversible condensation of oxaloacetate to the acetyl group of acetyl-CoA, is a common marker of mitochondrial content [14]. Reportedly, reduced citrate synthase activity has been shown to be associated with non-alcoholic fatty liver disease (NAFLD) [15]. These studies indicate the implications of mitochondrial dysfunction in the development of insulin resistance [2, 16].

Lipoprotein lipase (LPL) plays a major role in lipid metabolism via the hydrolysis of TG in chylomicrons and very-low-density lipoproteins [17, 18]. LPL is a rate-limiting enzyme to hydrolyze TG, one of the main components of lipid droplets, into glycerol and fatty acids. Produced free fatty acids are taken up and used for metabolic energy or storage as TG after re-esterization. LPL is mainly expressed in the adipose tissue and muscle and then translocated to the luminal surface of vascular endothelial cells [18, 19]. LPL expression in the liver is relatively high at birth. A previous study showed that LPL expression is elevated in hepatic stellate cells in patients with NAFLD [20]; however, LPL is barely expressed in the normal adult liver [21, 22]. Therefore, liver-specific overexpression of LPL is expected to hydrolyze TG in the liver, leading to suppression of hepatic lipid accumulation.

In this study, we aimed to determine the effects of liver-specific overexpression of LPL on hepatic lipid accumulation and metabolism using an adenovirus (Ad) vector. We hypothesize that liver-specific LPL overexpression suppresses hepatic lipid accumulation, leading to the improvement of insulin resistance. Systemic administration of Ad vectors results in the liver-specific expression of exogenous genes [23], whereas the main adverse effect of Ad vectors is hepatotoxicity [24, 25]. To reduce Ad vector-induced hepatotoxicity, we utilized a modified Ad vector named Ad-E4-122aT [26, 27] with higher and longer-term transgene expression and lower hepatotoxicity than that obtained using conventional Ad vectors. This enabled us to investigate the effect of hepatic LPL overexpression on high-fat diet (HFD)-induced lipid accumulation and glucose metabolism. Our findings may provide insights into novel therapeutic targets for treating glucose metabolism.

## Materials and methods

### Mice

Six-week-old male C57BL/6 mice were obtained from Nippon SLC (Hamamatsu, Japan). A normal diet (ND) (CE-2) and an HFD (High Fat Diet 32, 56.7% kcal from fat) were obtained from CLEA Japan (Tokyo, Japan). The mice were maintained on a 12/12 h light/dark cycle at 23°C and provided ad libitum access to water and food. C57BL/6 mice were intravenously treated with Ad vectors at a dose of 5 × 10^9^ infectious units (IFU)/mouse via the tail vein and were simultaneously fed an HFD. PBS-treated mice were fed ND through the experiments. The mice were anesthetized using 3% isoflurane (Pfizer, Tokyo, Japan) and euthanized by cervical dislocations. No adverse effects were observed in the experimental animals. Body weight was measured weekly. All animal procedures were approved by the Institutional Animal Care and Use Committee of Osaka Ohtani University (approval IDs 1407 and 2002) and were performed in accordance with the institutional guidelines and regulations for animal experiments at Osaka Ohtani University. All efforts were made to minimize animal suffering.

### Plasmids and Ad vectors

Ad vectors were constructed using an improved *in vitro* ligation method [28, 29]. LPL cDNA was amplified via polymerase chain reaction (PCR) using cDNA from C57BL/6 mouse liver as a template. The CA promoter (a fusion promoter comprising chicken β-actin promoter and cytomegalovirus enhancer)-driven LPL expression plasmid, pHMCA-LPL, was constructed using the PCR fragment and pHMCA6 [30]. pHMCA-LPL was sequenced and digested using I-CeuI/PI-SceI. The pHMCA-LPL fragment was ligated to the I-CeuI/PI-SceI-digested Ad vector plasmid pAdHM4-E4-122aT [26], yielding pAd-LPL. pAd-Luc, a firefly luciferase-expressing plasmid, was used as a control [31]. All Ad vectors (Ad-LPL and Ad-Luc) were purified as previously described [26]. Virus particles were evaluated using a previously described spectrophotometric method [32], and biological titers were determined using an Adeno-X-rapid titer kit (Clontech, Mountain View, CA, USA) according to the manufacturer’s instructions. The ratio of the particle-to-biological titer was between 6.0 and 7.2 for each Ad vector used in this study.

### Quantitative reverse transcription PCR (RT-PCR) analysis of gene expression

Total RNA was extracted from mouse livers using TRIzol (Thermo Fisher Scientific, Waltham, MA, USA) following the manufacturer’s instructions. cDNA was subsequently synthesized using the SuperScript VILO Master Mix kit (Thermo Fisher Scientific). The mRNA levels of the genes encoding LPL, PPARα, CPT1, acyl-CoA oxidase 1 (ACOX1), citrate synthase, NADH: ubiquinone oxidoreductase subunit AB1 (NDUFAB1), CPT2, and β-actin were determined via quantitative RT-PCR performed using THUNDERBIRD SYBR qPCR Mix (TOYOBO, Osaka, Japan), and mRNA levels were normalized to β-actin mRNA. The following thermal cycling protocol was used: 60 s at 95°C, followed by 40 cycles of 15 s at 95°C and 60 s at 63°C. Primer sequences are provided in S1 Table.

### Western blot analysis

Liver tissues were homogenized in RIPA buffer (Thermo Fisher Scientific) containing 5 mM dithiothreitol, 200 mM phenylmethylsulfonyl fluoride, protease inhibitor cocktail (Sigma-Aldrich, St. Louis, MO, USA), 5 mM EDTA, 10 mM NaF, and 10 mM Na_3_VO_4_. After sonication, liver proteins were separated via sodium dodecyl sulfate-polyacrylamide gel electrophoresis and electro-transferred to membranes using a Trans-Blot Turbo system (Bio-Rad, Hercules, CA, USA). After blocking with Blocking One (Nacalai Tesque, Kyoto, Japan), the membrane was incubated with rabbit anti-LPL antibody (1:1000; catalog no. GTX101125; GeneTex, Irvine, CA, USA), anti-CPT1 antibody (1:4000; catalog no. 15184-1-AP; Proteintech Group Inc, Rosemont, IL, USA), anti-ACOX1 antibody (1:4000; catalog no. 10957-1-AP; Proteintech), anti-citrate synthase antibody (1:4000; catalog no. 16131-1-AP; Proteintech), or mouse anti-β-actin antibody (1:5000; catalog no. A5441; Sigma-Aldrich; 1:5000; catalog no. 60008-1-Ig; Proteintech), followed by incubation with horseradish peroxidase-labeled anti-rabbit antibody (1:5000; catalog no. #7074) or anti-mouse IgG antibody (1:5000, catalog no. #7076; Cell Signaling Technology, Danvers, MA, USA). The western blot results were analyzed using the NIH image analysis software ImageJ (NIH, Bethesda, MD, USA). The protein levels were normalized to that of β-actin.

### Histological analysis

For histopathological examination of mouse tissues, tissue samples were collected, washed with phosphate-buffered saline, and fixed in 10% buffered formalin. The samples were embedded in paraffin, sectioned (2–3 μm thick), and stained with hematoxylin and eosin at Applied Medical Research (Osaka, Japan).

For Oil Red O staining, the mouse tissue samples were washed with phosphate-buffered saline and fixed in 4% paraformaldehyde. Frozen sections were prepared and stained with an Oil Red O staining solution at Applied Medical Research. For semi-quantitative histopathological comparison, each section was analyzed using the NIH image analysis software ImageJ.

For transmission electron microscopy analysis, liver samples were fixed in half Karnovsky fixative solution and washed with cacodylate buffer. Sample preparation, photographing, measurement of mitochondrial areas and counting of mitochondria numbers were performed by Applied Medical Research. The area of mitochondria was determined by measuring the area of the mitochondria in the cell.

### Serum TG and free fatty acid analysis

Under anesthesia, blood samples were collected via retro-orbital bleeding, and serum was obtained by centrifuging. Serum TG and free fatty acid levels were determined using LabAssay Triglyceride and LabAssay NEFA kits (FUJIFILM Wako Pure Chemical Corporation, Osaka, Japan), respectively, according to manufacturers’ instructions.

### Blood glucose analysis

After 2 weeks of Ad vector administration, the mice were fasted for 16 h. Afterward, the mice were intraperitoneally injected with glucose (1.5 g/kg). Blood glucose levels were determined immediately before and at the indicated time after injection using Glutest Sensor Neo (Sanwa Kagaku Kenkyusho, Nagoya, Japan).

### Insulin tolerance tests (ITTs)

After 2 weeks of Ad vector administration, the mice were fasted for 6 h. Afterward, the mice were intraperitoneally injected with insulin (0.75 units/kg). Blood glucose levels were determined using Glutest Sensor Neo immediately before and at an indicated time after injection.

### Assessment of fasting serum insulin levels

Under anesthesia, blood samples were collected via retro-orbital bleeding from mice fasted for 16 h after 2 weeks of Ad vector administration, and serum was obtained by centrifuging the samples. An ELISA kit (Mercodia, Uppsala, Sweden) was used to measure serum insulin levels in accordance with the manufacturer’s instructions. The homeostasis model assessment of insulin resistance (HOMA-IR) value was calculated as follows: fasting glucose (mg/dL) × fasting insulin (μIU/mL)/405.

### Statistical analysis

Statistical analysis was performed using BellCurve for Excel (Social Survey Research Information Co., Ltd., Tokyo, Japan). The Mann–Whitney *U* test was used to compare the differences between two independent groups, and one-way ANOVA with Dunnett’s post hoc tests was used for multiple comparisons. Data are presented as mean ± standard error, and statistical significance was considered at p < 0.05.

## Results

### Hepatic LPL overexpression attenuated lipid accumulation in the liver

**Fig 1:**
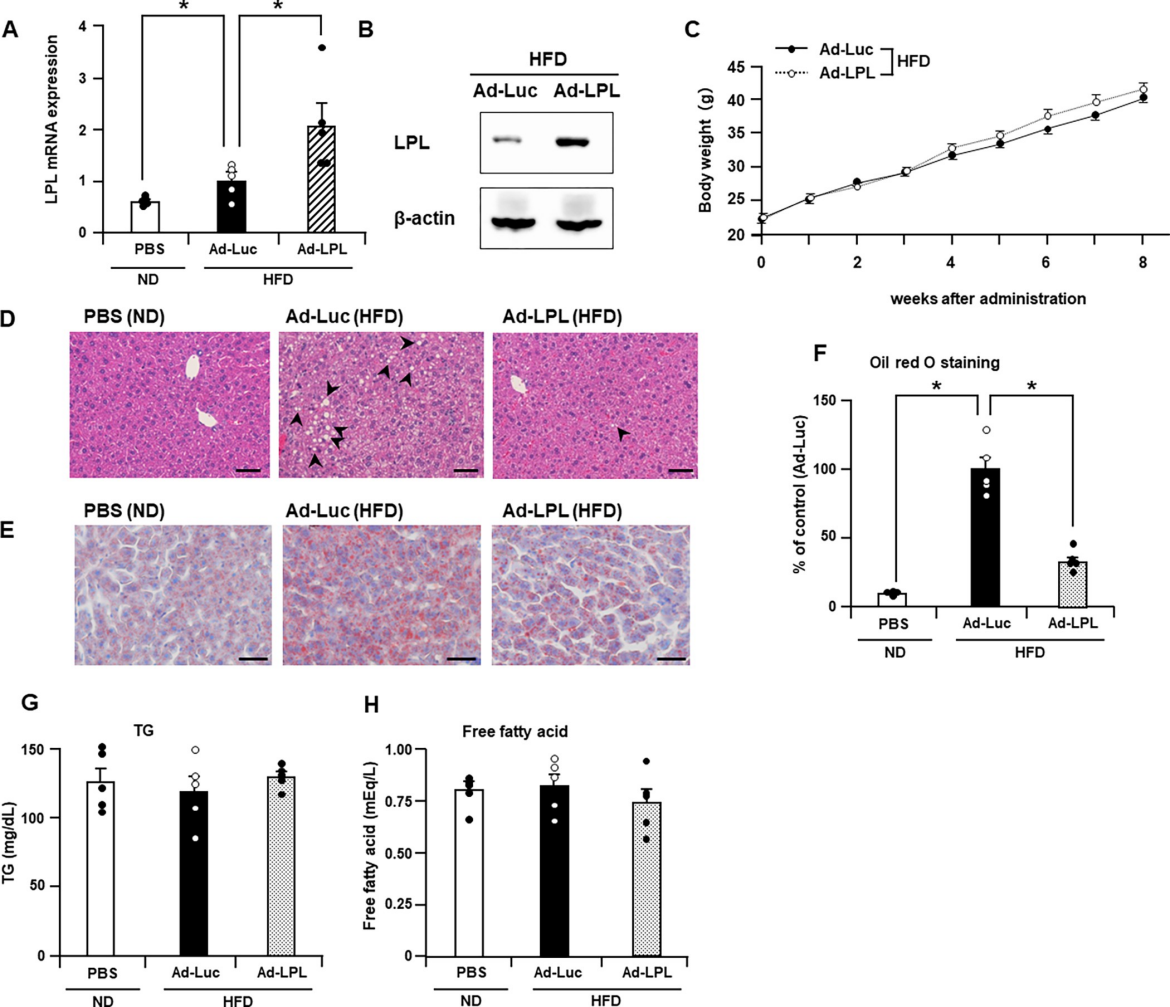
Hepatic LPL overexpression attenuates lipid accumulation in high-fat diet (HFD)-fed mice liver. Male C57BL/6 mice were intravenously treated with Ad vectors at a dose of 5 × 10^9^ IFU/mouse via the tail vein and were simultaneously fed HFD. PBS-treated mice were fed ND throughout the experimental period. Hepatic LPL (A) mRNA and (B) protein levels determined using quantitative RT-PCR and western blot analysis, respectively, 2 weeks after administration of Ad-LPL, Ad-Luc, or PBS. Hepatic LPL mRNA levels in Ad-Luc-treated mice were set as 1.0. (C) Body weights of mice determined weekly. Liver sections obtained 2 weeks after administration of Ad-LPL, Ad-Luc, or PBS and stained with (D) hematoxylin and eosin, or (E) Oil red O. Arrowheads indicate lipid droplets. Bar = 50 μm. (F) Semi-quantitative analysis of Oil red O staining performed using ImageJ. (G) Fasting serum triglyceride and (H) free fatty acid levels in mice 2 weeks after Ad vector treatment. One-way ANOVA with Dunnett’s post hoc tests was used for multiple comparisons (A, F–H). The Mann–Whitney *U* test was used to compare the differences between two independent groups (C). The data are expressed as the mean ± standard error (n = 5). *, P < 0.05 vs. Ad-Luc. ND, normal diet; HFD, high-fat diet.

To overexpress LPL in the mouse liver, C57BL/6 mice were intravenously injected with Ad-LPL or Ad-Luc as the control Ad vector and simultaneously fed HFD. Two weeks after administration of Ad vectors, the hepatic LPL mRNA level in Ad-LPL-treated mice was 2-fold higher than that in Ad-Luc-treated mice (Fig 1A). LPL protein levels were considerably higher in mouse liver tissues administered with Ad-LPL than that in Ad-Luc-administered mouse liver tissues (Fig 1B). To examine whether intravenous administration of Ad-LPL leads to liver-specific overexpression of LPL in mice, LPL mRNA levels in the epididymal adipose tissue and skeletal muscle were determined using quantitative RT-PCR. LPL mRNA and protein levels in epididymal adipose tissue and skeletal muscle were similar in the Ad-LPL and Ad-Luc-treated mice (S1A and S1B Fig). In addition, no significant difference in the serum LPL activity levels was observed between Ad-LPL- and Ad-Luc-treated mice (S1C Fig). These results indicate that intravenous administration of Ad-LPL leads to overexpression of LPL in a liver-specific manner. The body weights of the mice were monitored for 8 weeks. The increase in body weight was similar in the Ad-LPL- and Ad-Luc-treated mice (Fig 1C).

To investigate the effect of LPL overexpression on lipid accumulation in the liver, we performed a histopathological examination of liver sections via hematoxylin and eosin staining. Many lipid droplets were observed in the livers of mice treated with Ad-Luc. Lipid droplet formation was considerably decreased in Ad-LPL-treated mice compared to that in Ad-Luc-treated mice (Fig 1D). Oil Red O staining of liver sections also revealed a lower amount of lipid droplets in Ad-LPL-treated mice than in Ad-Luc-treated mice (Fig 1E and 1F). No lipid droplet formation was observed in the skeletal muscle of Ad-LPL and Ad-Luc-treated mice (S1D Fig). No significant difference in fasting serum TG and free fatty acid levels was observed between the Ad-LPL- and Ad-Luc-treated mice (Fig 1G and 1H). These results indicate that LPL overexpression in the liver can suppress hepatic lipid accumulation without altering serum TG and free fatty acid levels.

### Hepatic LPL overexpression improved glucose metabolism and insulin resistance

**Fig 2:**
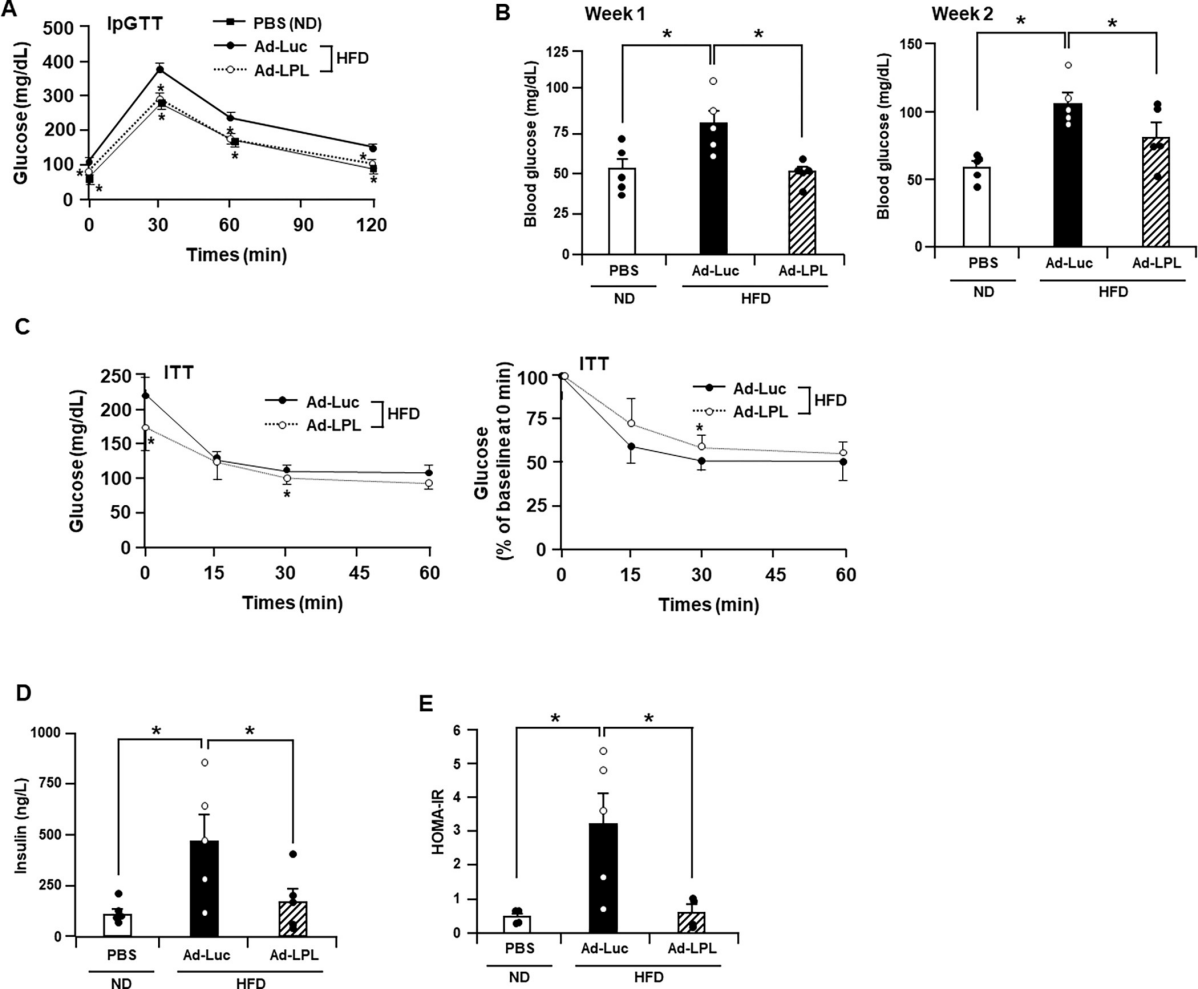
Hepatic LPL overexpression improves glucose metabolism and insulin resistance in HFD-fed mice. Male C57BL/6 mice were intravenously treated with Ad vectors via the tail vein and were simultaneously fed HFD. PBS-treated mice were fed ND throughout the experimental period. (A) Blood glucose levels during the intra-peritoneal glucose tolerance test of mice 2 weeks after administration of Ad-LPL, Ad-Luc, or PBS. (B) Fasting blood glucose levels of mice 1 and 2 weeks after administration of Ad-LPL, Ad-Luc, or PBS. (C) Blood glucose levels during the intra-peritoneal insulin tolerance test of mice 2 weeks after administration of Ad-LPL, Ad-Luc, or PBS. (D) Fasting insulin levels and (E) HOMA-IR of mice 2 weeks after administration of Ad-LPL, Ad-Luc, or PBS. One-way ANOVA with Dunnett’s post hoc tests was used for multiple comparisons (A, B, D, and E). The Mann–Whitney *U* test was used to compare differences between two independent groups (C). The data are expressed as the mean ± standard error (n = 4–5). *, P < 0.05 in comparison with Ad-Luc.

To determine the effect of LPL overexpression on glucose metabolism in a mouse model of HFD-induced insulin resistance, we performed a glucose tolerance test two weeks after Ad vector administration. Glucose levels at 30, 60, and 120 min after glucose injection were significantly lower in Ad-LPL-treated mice than that in Ad-Luc-treated mice (Fig 2A). One and two weeks after the administration of Ad vectors, the fasting blood glucose levels were considerably lower in Ad-LPL-treated mice than in Ad-Luc-treated mice (Fig 2B). Next, we performed the ITT 2 weeks after the administration of Ad vectors. Blood glucose levels and the rates of decrease in blood glucose levels against 0 min of insulin injection of insulin to Ad-LPL-treated mice were approximately similar to those of Ad-Luc mice that received the same treatment (Fig 2C).

To investigate the effect of LPL overexpression on insulin resistance, fasting insulin levels in mice were determined two weeks after the administration of Ad vectors. Fasting serum insulin levels were considerably lower in the Ad-LPL-treated mice than that in the Ad-Luc-treated mice (Fig 2D). HOMA-IR, an index of insulin resistance, was also considerably lower in mice administered with Ad-LPL than in those administered with Ad-Luc (Fig 2E). These results suggest that LPL overexpression in the liver improves hepatic glucose metabolism and insulin resistance and does not alter insulin resistance in the skeletal muscle.

### Hepatic LPL overexpression upregulated fatty acid oxidation-related genes and proteins

**Fig 3:**
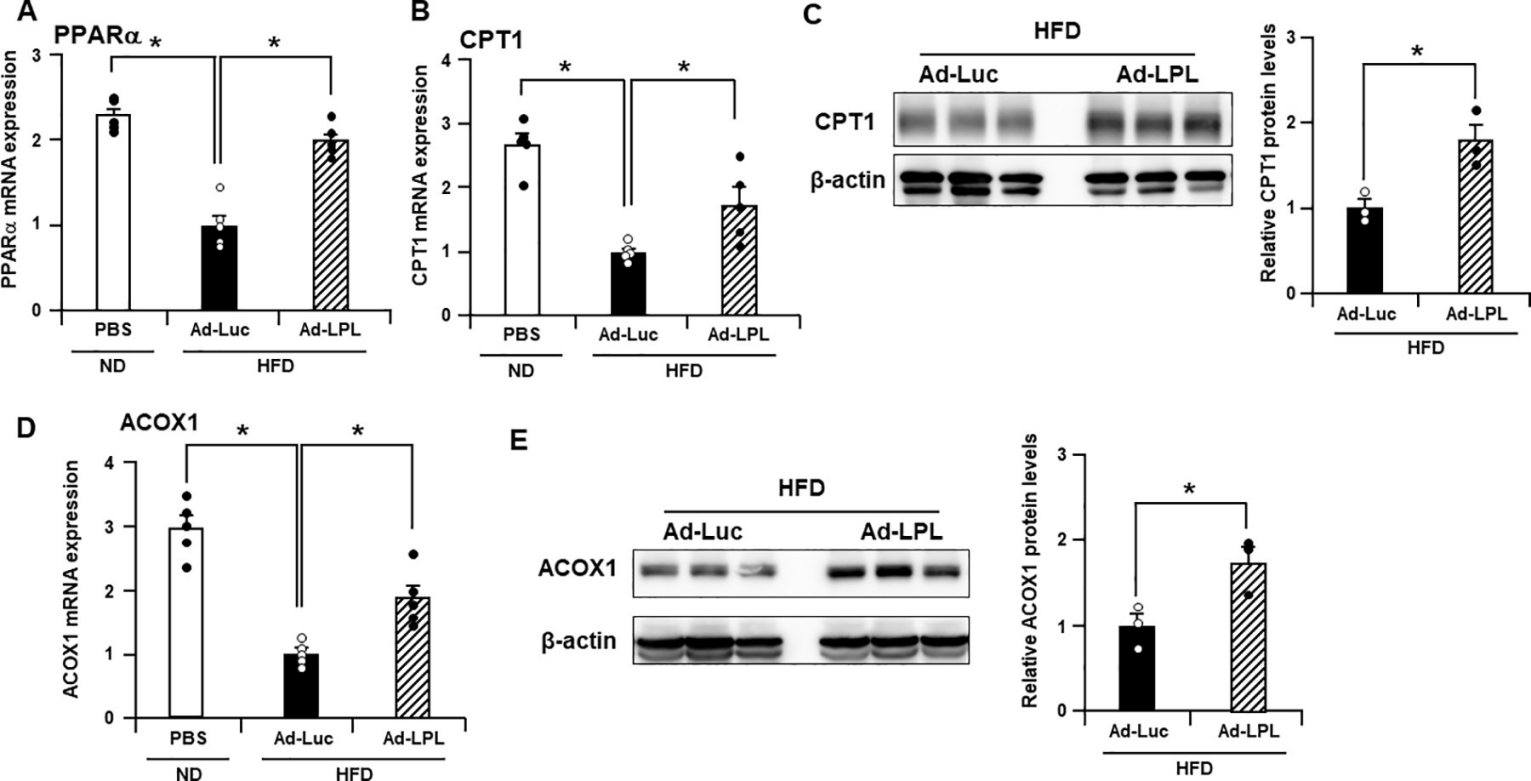
Hepatic LPL overexpression upregulates fatty acid oxidation-related genes and protein in HFD-fed mice. Male C57BL/6 mice were intravenously treated with Ad vectors via the tail vein and were simultaneously fed HFD. PBS-treated mice were fed ND throughout the experimental period. Hepatic (A) PPARα, (B) CPT1, and (D) ACOX1 mRNA levels in male C57BL/6 mice 2 weeks after administration of Ad-LPL, Ad-Luc, or PBS determined using quantitative RT-PCR. The data were normalized to the corresponding value in the Ad-Luc-treated group. One-way ANOVA with Dunnett’s post hoc tests was used for multiple comparisons. The data are expressed as the mean ± standard error (n = 5). Hepatic (C) CPT1 and (E) ACOX1 protein levels in male C57BL/6 mice 2 weeks after administration of Ad-LPL or Ad-Luc determined using western blot analysis. The quantification data were normalized to the corresponding value in the Ad-Luc-treated group. The Mann–Whitney *U* test was used to compare the differences between two independent groups. The data are expressed as the mean ± standard error (n = 3). *, P < 0.05 vs. Ad-Luc.

Hepatic LPL overexpression in HFD-fed mice was expected to alter gene expression in the liver. Hepatic overexpression of LPL, which hydrolyzes TG into fatty acids and glycerol, attenuated hepatic lipid accumulation (Fig 1D and 1E); however, serum TG and free fatty acids levels did not change in Ad-Luc and Ad-LPL-treated mice (Fig 1G and 1H). Fatty acids can be degraded by oxidation. Therefore, we focused on the fatty acid oxidation-related genes in the mouse liver. PPARα and CPT1 mRNA levels in the livers of Ad-Luc-treated mice fed HFD were 2.3–2.7-fold lower than those in the livers of PBS-treated mice fed ND (Fig 3A and 3B). PPARα and CPT1 mRNA levels increased 1.7–2.0-fold in the livers of Ad-LPL-treated mice compared to those in Ad-Luc-treated mice. CPT1 protein expression was also higher in Ad-LPL-treated mouse liver than in the Ad-Luc-treated mouse liver (Fig 3C). Hepatic mRNA levels of ACOX1, a peroxisomal fatty-acid oxidation enzyme [33], in Ad-Luc-treated mice were 3.0-fold lower than those in PBS-treated mice fed ND (Fig 3D). ACOX1 mRNA expression in Ad-LPL-treated mouse liver was 1.9-fold higher than that in Ad-Luc-treated mouse liver. Hepatic ACOX1 protein expression was also higher in Ad-LPL-treated mice than in Ad-Luc-treated mice (Fig 3E). These results indicate that hepatic LPL overexpression upregulates fatty acid oxidation-related genes and proteins in the liver.

### Hepatic LPL overexpression maintained mitochondrial content

**Fig 4:**
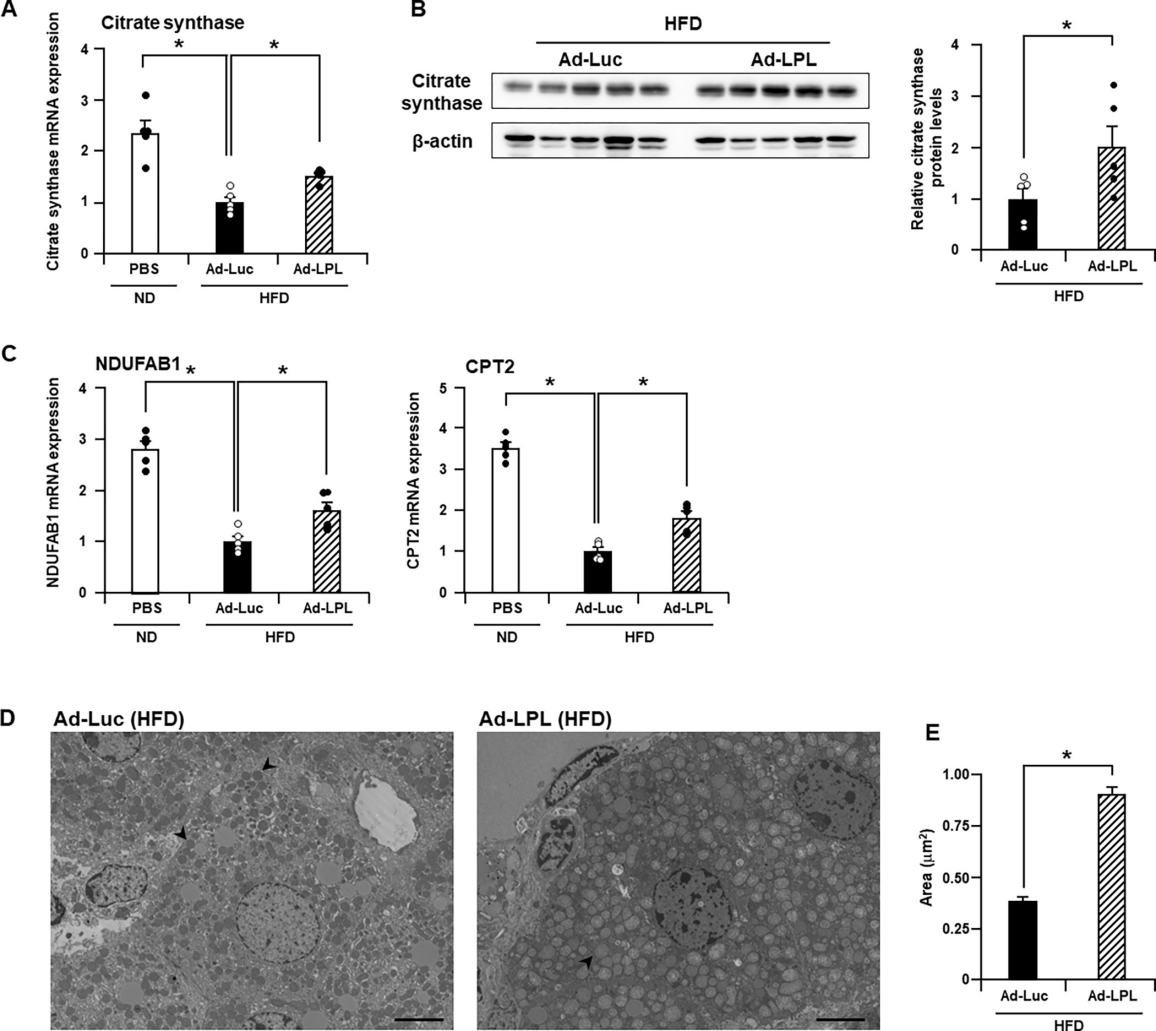
Hepatic LPL overexpression maintains mitochondrial content in HFD-fed mice. Male C57BL/6 mice were intravenously treated with Ad vectors via the tail vein and were simultaneously fed HFD. PBS-treated mice were fed ND throughout the experimental period. Two weeks after administration of Ad-LPL, Ad-Luc, or PBS, (A, C) expression of mitochondrial genes in the liver determined using quantitative RT-PCR. The data were normalized to the corresponding values in the Ad-Luc-treated group. One-way ANOVA with Dunnett’s post hoc tests was used for multiple comparisons. Hepatic (B) citrate synthase protein levels in male C57BL/6 mice 2 weeks after administration of Ad-LPL or Ad-Luc determined using western blot analysis. The quantification data were normalized to the corresponding value in the Ad-Luc-treated group. The Mann–Whitney *U* test was used to compare the differences between two independent groups. (D) The area of mitochondria in the liver was assessed via transmission electron microscopy analysis. Arrowheads indicate mitochondria. Bar = 5 μm. (E) The area of mitochondria was determined by measuring the area of mitochondria in the cell using Fig 4D. The Mann–Whitney *U* test was used to compare the differences between two independent groups. The data are expressed as the mean ± standard error (A–C: n = 5, E: n = 151–155). *, P < 0.05 vs. Ad-Luc.

As mitochondria play a crucial role in fatty acid oxidation, we assessed the expression of the mRNA and proteins of mitochondrial genes using quantitative RT-PCR and western blot analysis, respectively, and the area of mitochondria using transmission electron microscopy. The citrate synthase mRNA level in the livers of Ad-Luc-treated mice fed HFD was 2.4-fold lower than that in the livers of PBS-treated mice fed ND. On the contrary, it was 1.5-fold higher in the livers of Ad-LPL-treated mice than that in those of the Ad-Luc-treated mice (Fig 4A). Citrate synthase protein expression in Ad-LPL-treated mouse liver was also higher than that in Ad-Luc-treated mouse liver (Fig 4B). The mRNA levels of hepatic NDUFAB1 and CPT2 were also higher in Ad-LPL-treated mice than those in Ad-Luc-treated mice (Fig 4C). Transmission electron microscopy analysis followed by subsequent measurement of the area of individual mitochondria revealed a 2.3-fold smaller area of mitochondria in the Ad-Luc-treated mouse liver than that in the Ad-LPL-treated mouse liver (Fig 4D and 4E). These results suggest that hepatic LPL overexpression partially maintains the mitochondrial content.

## Discussion

LPL is a key enzyme responsible for the hydrolysis of circulating TGs and is mainly expressed in extrahepatic tissues, such as the adipose tissue and muscle. The potential effects of hepatic LPL overexpression on lipid accumulation in the liver and metabolism in mice fed an HFD are unknown. In this study, we focused on the potential of LPL to suppress lipid accumulation in the liver. C57BL/6 mice were administered with Ad-LPL to induce LPL overexpression in the liver and simultaneously fed an HFD. Hepatic LPL overexpression suppressed lipid accumulation in the liver and improved glucose metabolism and insulin resistance.

Attenuation of hepatic lipid accumulation by LPL overexpression in the liver was expected to alter serum TG and fatty acid levels; however, the levels of serum TG and free fatty acids were similar in Ad-Luc and Ad-LPL-treated mice. This result indicated that TG degradation in the liver by this system was liver-specific rather than affecting the amount of TG and free fatty acid in the blood. Ectopic lipids, intramyocellular or in the liver, are associated with insulin resistance. It has been shown that intramyocellular TG content is a stronger predictor of muscle insulin resistance than circulating fatty acids [34]. In this study, the levels of PPARα, CPT-1, and ACOX1, which are upregulated with activation of fatty acid oxidation [9–11], were higher in the Ad-LPL-treated mice than in the Ad-Luc-treated mice. Therefore, it can be inferred that liver-specific LPL overexpression increases fatty acid oxidation.

An important function of insulin in the liver involves suppression of glucose production. Hepatic insulin resistance impairs gluconeogenesis suppression and contributes to high fasting blood glucose levels [35, 36]. Under HFD-fed conditions, hepatic gluconeogenesis is activated, resulting in hyperglycemia and high insulin levels stimulating liver fatty acid synthesis from glucose and TG formation [37]. Our study findings indicate that hepatic LPL overexpression can ameliorate HFD-induced lipid accumulation in the liver without altering the systemic levels of TG and free fatty acids, leading to improvement in glucose tolerance, fasting blood glucose, and insulin resistance, as assessed by HOMA-IR. Although the molecular mechanism by which lipid causes insulin resistance is not fully elucidated, numerous experimental and clinical studies have shown a close correlation between insulin sensitivity and ectopic lipid storage in the muscle and liver [34, 36, 38]. Hepatic lipids, such as diacylglycerol, are associated with the activation of protein kinase Cε, leading to impairment of insulin signaling [36, 39]. Liver-specific overexpression of LPL may hydrolyze diacylglycerol and suppress diacylglycerol-mediated activation of protein kinase Cε and subsequent impairment of insulin signaling. Therefore, decreasing ectopic lipid content in non-adipose tissue by promoting its tissue oxidation would improve insulin sensitivity. Nevertheless, further research is needed to confirm these speculations and elucidate the precise mechanism underlying the association between lipid accumulation and insulin resistance.

LPL functions as a key enzyme for the generation of PPARα ligands via the hydrolysis of very-low-density lipoprotein and PPARα activation [40]. LPL hydrolyzes TG to generate free fatty acids. The expression of fatty acid oxidation-related genes, such as PPARα and ACOX1, increased in Ad-LPL-treated mice than that in Ad-Luc-treated mice. These results indicated that free fatty acids produced by LPL may correlate with an increased expression of fatty acid oxidation-related genes and activate fatty acid oxidation.

In this study, overexpression of hepatic LPL upregulated the fatty acid oxidation-related genes and proteins in the liver of Ad vector-administered mice. The mRNA and protein levels of citrate synthase, the most common marker of mitochondrial content, and the area of mitochondria in HFD-fed Ad-Luc-treated mouse livers were lower than that in HFD-fed Ad-LPL-treated mouse livers. NDUFAB1, a mitochondrial acyl carrier protein, is an essential component of the mitochondrial fatty acid synthesis pathway, and its knockdown in 293T cells reduced complex 1 activity [41]. CPT2, localized at the matrix of the mitochondrial inner membrane, regenerates acyl-CoA, entering the fatty acid oxidation pathway [11]. HFD-fed Ad-LPL-treated mice showed increased expression of genes involved in fatty acid oxidation (CPT1 and CPT2) and NDUFAB1 compared to Ad-Luc-treated mice. Mitochondria play an important role in cellular metabolism via fatty acid oxidation during ATP production. Mitochondrial dysfunction is closely associated with the development of insulin resistance, leading to type 2 diabetes mellitus [2, 16, 42]. Reduced mitochondrial enzyme activity and lipid oxidation have been observed in the skeletal muscles of individuals with obesity and insulin resistance [12, 43]. The size of mitochondria and activity of marker enzymes of the oxidative pathway have also been reduced in individuals with obesity and type 2 diabetes mellitus. They correlate with the degree of insulin resistance [13]. Taken together, attenuation of lipid accumulation by Ad vector-mediated liver-specific overexpression of LPL in the liver may contribute to maintaining mitochondrial function and improving insulin resistance.

In contrast to our findings, a previous study has reported that standard chow-fed transgenic mice with liver-specific overexpression of LPL exhibit increased hepatic lipid accumulation and insulin resistance [44]. This discrepancy could be attributed to the manner of LPL overexpression and mouse feeding. Kim *et al*. generated standard mouse chow-fed transgenic mice with liver-specific LPL overexpression. In our study, LPL-overexpressing C57BL/6 mice were established using the Ad vector and fat-feeding protocols. Nevertheless, some studies have provided evidence that increased LPL activity may be potentially beneficial, similar to our study [45, 46].

In summary, this study showed that LPL overexpression in the livers of HFD-fed mice suppressed hepatic lipid accumulation and improved glucose metabolism. LPL may represent a new therapeutic target for treating glucose metabolism disorders, including type 2 diabetes mellitus and hepatic steatosis, such as non-alcoholic fatty liver disease.

## Supporting information

S1 Table Primer sequences used for quantitative RT-PCR. (DOCX)

S1 Text Supplemental methods. (DOCX)

S1 Fig (TIF)
