# Supplementary material for: Liver-specific overexpression of lipoprotein lipase improves glucose metabolism in high-fat diet-fed mice
Source: PLoS One. 2022 Sep 13;17(9):e0274297. doi: 10.1371/journal.pone.0274297 (PMC9469954; doi:10.1371/journal.pone.0274297)
Supplement: S1 Table — (DOCX) [file pone.0274297.s001.docx]

**S1 Table** Primer sequences used for quantitative RT-PCR

| **Gene** | **Forward primer (5′–3′)** | **Reverse primer (5′–3′)** |
| --- | --- | --- |
| LPL | ATGGATGGACGGTAACGGGAA | CCCGATACAACCAGTCTACTACA |
| PPARα | AACATCGAGTGTCGAATATGTGG | CCGAATAGTTCGCCGAAAGAA |
| CPT1 | CTCCGCCTGAGCCATGAAG | CACCAGTGATGATGCCATTCT |
| ACOX1 | ATGGGTCATGGAACTCATCT | ACCACTTGATGGAAGTCACA |
| citrate synthase | GGACAATTTTCCAACCAATCTGC | TCGGTTCATTCCCTCTGCATA |
| NDUFAB1 | TTTGTGCCGCCAGTACAGTG | TCAAACCCAAATTCGTCTTCCA |
| CPT2 | CAAAAGACTCATCCGCTTTGTTC | CATCACGACTGGGTTTGGGTA |
| β-actin | GGCTGTATTCCCCTCCATCG | CCAGTTGGTAACAATGCCATGT |
| β-tubulin | CAACTTCGTTTTCGGTCAGTCT | GACAGAGTCAACCAACTCAGC |
