# Supplementary material for: Liver-specific overexpression of lipoprotein lipase improves glucose metabolism in high-fat diet-fed mice
Source: PLoS One. 2022 Sep 13;17(9):e0274297. doi: 10.1371/journal.pone.0274297 (PMC9469954; doi:10.1371/journal.pone.0274297)
Supplement: S1 Text — (DOCX) [file pone.0274297.s002.docx]

**S1 Text**

**Supplementary methods**

**Quantitative reverse transcription PCR (RT-PCR) analysis of gene expression in adipose tissue and skeletal muscle**

The LPL mRNA levels in the epididymal adipose tissue and skeletal muscle were determined via quantitative RT-PCR performed using THUNDERBIRD SYBR qPCR Mix. mRNA levels of the adipose tissue and skeletal muscle were normalized to β-actin and β-tubulin mRNA, respectively. Primer sequences are provided in S1 Table.

**Western blot analysis of adipose tissue and skeletal muscle**

Epididymal adipose tissue and skeletal muscle proteins were separated via sodium dodecyl sulphate-polyacrylamide gel electrophoresis and electro-transferred to membranes. After blocking, the membrane was incubated with rabbit anti-LPL antibody, mouse anti-β-actin antibody, or rabbit anti-β-tubulin antibody (1:500; catalog no. ab6046; abcam, Cambridge, United Kingdom) followed by incubation with horseradish peroxidase-labeled anti-rabbit antibody or anti-mouse IgG antibody.

**LPL activity assay**

Serum LPL activity was determined using Lipoprotein Lipase Activity Assay Kit (Fluorometric; Cell Biolabs, Inc., San Diego, CA, USA).


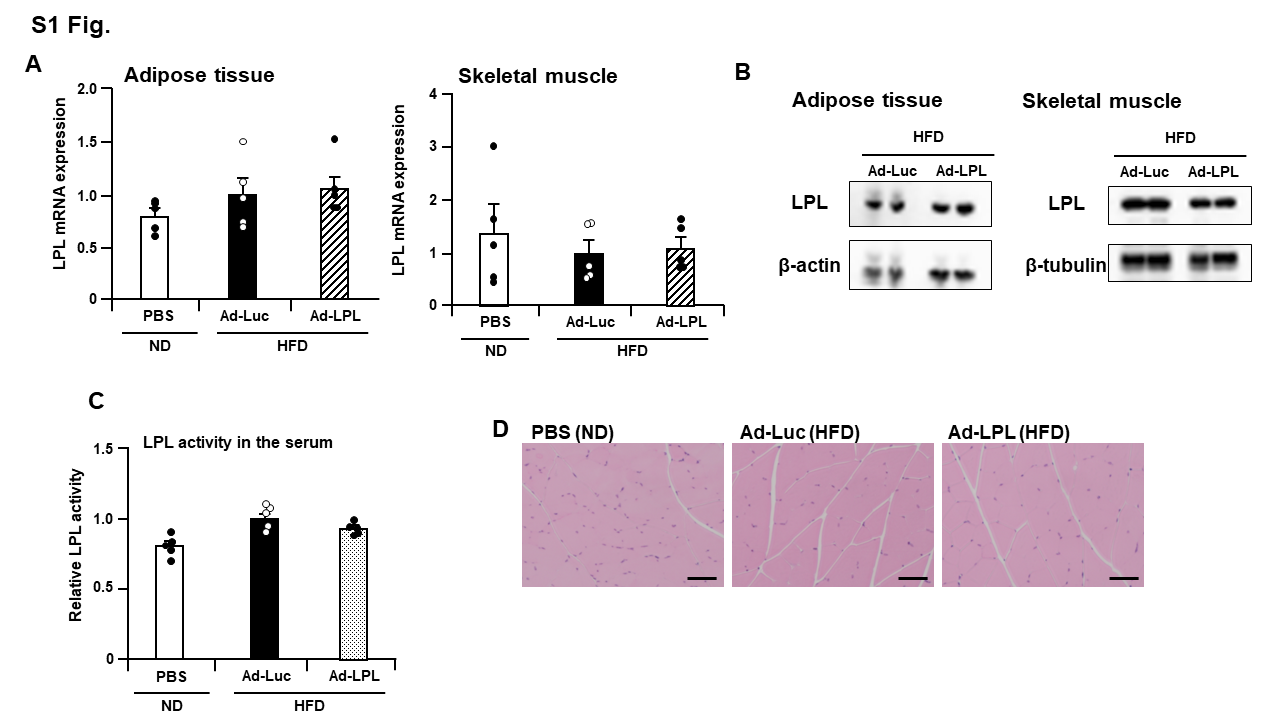


**Fig S1. Intravenous administration of Ad-LPL did not affect LPL expression in adipose tissue and skeletal muscle.**

Male C57BL/6 mice were intravenously treated with Ad vectors at a dose of 5 × 10^9^ IFU/mouse via the tail vein and were simultaneously fed HFD. PBS-treated mice were fed ND throughout the experimental period. Two weeks after administration of Ad-LPL, Ad-Luc, or PBS, (A) LPL mRNA levels of adipose tissue and skeletal muscle determined using quantitative RT-PCR. mRNA levels in Ad-Luc-treated mice were set as 1.0. (B) LPL protein levels of adipose tissue and skeletal muscle determined using western blot analysis. (C) LPL activity levels in the serum determined using LPL activity assay kit. Skeletal muscle sections were stained with (D) hematoxylin and eosin staining. Bar=50 μm. One-way ANOVA with Dunnett’s post hoc tests was used for multiple comparisons. The data are expressed as the mean ± standard error (n = 5). ND, normal diet; HFD, high-fat diet.
