# Supplementary figures and images for: Liver-specific overexpression of lipoprotein lipase improves glucose metabolism in high-fat diet-fed mice
Source: PLoS One. 2022 Sep 13;17(9):e0274297. doi: 10.1371/journal.pone.0274297 (PMC9469954; doi:10.1371/journal.pone.0274297)

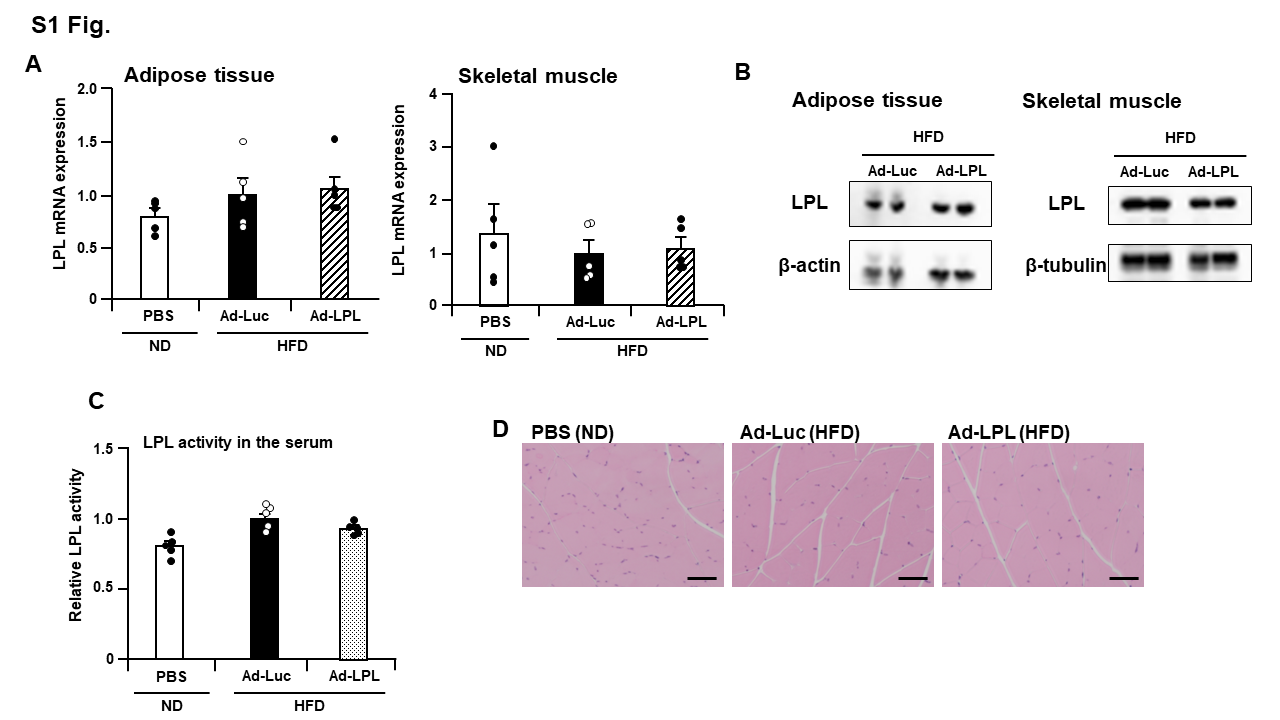

Supplement: S1 Fig — (TIF) [file pone.0274297.s003.TIF]
